# A Study on Survival Analysis Methods Using Neural Network to Prevent Cancers

**DOI:** 10.3390/cancers15194757

**Published:** 2023-09-27

**Authors:** Chul-Young Bae, Bo-Seon Kim, Sun-Ha Jee, Jong-Hoon Lee, Ngoc-Dung Nguyen

**Affiliations:** 1 Mediage Research Center, Seongnam-si 13449, Republic of Korea; 2 Department of Epidemiology and Health Promotion, Institute for Health Promotion, Graduate School of Public Health, Yonsei University, Seoul 03722, Republic of Korea; 3 Moadata AI Labs, Seongnam-si 13449, Republic of Korea

**Keywords:** survival analysis, recurrent neural network, LSTM, Cox PH, biomarkers, cancer, cohort, follow-up

## Abstract

**Simple Summary:**

According to cancer statistics published in 2020, there were 19.3 million new cancer cases and almost 10.0 million cancer deaths worldwide. This suggests that cancer is one of the main health threats worldwide. Survival analysis, combining the exponential distribution with regression analysis, can predict the time when a specific event will occur. This nationwide follow-up study aims to present survival deep learning models to assist in the early identification of cancer incidence. Our models consistently achieved high performance in 10 types of cancer. By applying the techniques outlined in this paper, clinical biomarkers, demographic, and anthropometric data can be utilized to predict the risk of cancer occurrence.

**Abstract:**

**Background:** Cancer is one of the main global health threats. Early personalized prediction of cancer incidence is crucial for the population at risk. This study introduces a novel cancer prediction model based on modern recurrent survival deep learning algorithms. **Methods:** The study includes 160,407 participants from the blood-based cohort of the Korea Cancer Prevention Research-II Biobank, which has been ongoing since 2004. Data linkages were designed to ensure anonymity, and data collection was carried out through nationwide medical examinations. Predictive performance on ten cancer sites, evaluated using the concordance index (c-index), was compared among nDeep and its multitask variation, Cox proportional hazard (PH) regression, DeepSurv, and DeepHit. **Results:** Our models consistently achieved a c-index of over 0.8 for all ten cancers, with a peak of 0.8922 for lung cancer. They outperformed Cox PH regression and other survival deep neural networks. **Conclusion:** This study presents a survival deep learning model that demonstrates the highest predictive performance on censored health dataset, to the best of our knowledge. In the future, we plan to investigate the causal relationship between explanatory variables and cancer to reduce cancer incidence and mortality.

## 1. Introduction

Cancer stands as a leading cause of premature death in upper-middle- and high-income countries [1]. Specifically, female breast, lung, colorectal, prostate, stomach, and liver cancers account for the highest incidence and mortality rates [1,2]. Early personalized prediction of cancer incidence holds paramount importance for the population at risk. Substantial evidence regarding the risk factors of noncommunicable diseases, including cancer, has primarily been drawn from large-scale follow-up studies, with many of them employing survival analysis.

Survival analysis is centered on determining the probability of surpassing a certain period without encountering the event, as well as relevant outcomes. It has demonstrated its robustness in comparing risks among subgroups within the targeted population and has found applications in diverse fields, spanning from economics to healthcare [3]. Consequently, survival analysis has been utilized in the quest to identify factors associated with cancer and predict cancer survival [4].

The Cox proportional hazard (Cox PH) regression model [5] has emerged as a popular tool within the realm of survival analysis due to its interpretability and predictive ability. However, it rests upon strong assumptions regarding covariates. To mitigate these limitations, numerous models have been developed based on the Cox PH model, incorporating artificial intelligence techniques [4]. One such machine learning approach, the random survival forest, has gained prominence. Certain studies [6,7,8] were among the first to introduce a single hidden layer network for survival analysis. Building upon similar concepts, Katzman [9] further developed a deep survival network for personalized treatment recommendations. The predictive efficacy of survival networks was markedly enhanced by the application of multitask learning and residual connections, as proposed in the model known as DeepHit [10].

Recent research has suggested that survival neural networks outperform their machine learning and traditional statistical counterparts [10,11]. Leveraging data from a large-scale blood-based cohort, we conducted a comparative assessment of the performance of various survival deep networks, using Cox PH as the benchmark, to predict ten cancers that rank among the most common globally [2,12]. Furthermore, we introduced nDeep—a model that, to the best of our knowledge, achieves the highest possible performance on real censored health datasets. In an attempt to interpret the effects of those biomarkers in each model, feature importance was also calculated.

## 2. Materials and Methods

### 2.1. Subjects

A total of 160,407 observations (95,229 men and 62,169 women) were collected from a blood-based cohort within The Korean Cancer Prevention Study-II Biobank, an ongoing study initiated in 2004 through nationwide medical examinations [13]. Participants underwent annual follow-ups utilizing personal identification numbers, enabling linkage with the National Cancer Center registry, hospital admission records, and death registers. The integrity of all linkages was guaranteed with anonymity safeguards. During routine health check-ups, individuals diagnosed with cancer or other severe conditions (such as organ failure, diabetes, malignant hypertension), as well as those who died, were subsequently excluded from the study. 

### 2.2. Characteristics, Biomarkers, and Events

Anthropometry (height and weight) was recorded by InBody (Biospace, Cheonan-si, Korea) when participants wore light clothes. For waist circumference, the thinnest area between the iliac crest and the inferior part of the lowest rib was measured in an upright position. Forced vital capacity and forced expiratory volume in 1 second were recorded by an electronic spirometer in standing position, while the better results out of two records were taken.

Seated blood pressure was then taken by a registered nurse or technician using a sphygmomanometer after resting 5 min. Fasting blood glucose, total cholesterol, and other biomarkers were measured in the laboratory of the hospital by a COBAS INTEGRA 800 (Roche, Berlin, Germany) and a 7600 Analyzer (Hitachi, Tokyo, Japan). More details can be found at [13]. For our final analysis, these features were included as covariates:

- Demographic: AGE—age, years; SEX—sex, 1 as male and 2 as female.
- Metabolic characteristics: WT—weight, kilograms; HT—height, centimeters; and BMI—body mass index = weight/height^2^ (kg/m^2^); WC—waist circumference, centimeters; SBP—systolic blood pressure, mmHg; DBP—diastolic blood pressure, mmHg; CHO total cholesterol, mg/dL; HDL—high-density cholesterol, mg/dL; TG—triglycerides, mg/dL; LDL—low-density cholesterol, mg/dL; GLU—glucose, mg/dL; PP—pulse pressure = systolic blood pressure − diastolic blood pressure, mmHg.
- Liver function: ALB—albumin, g/dL; GLOBULIN—globulin, g/dL; AGR—albumin globulin rate; BIL—total bilirubin, mg/dL; DBIL—direct bilirubin, mg/dL; ALP—alkaline phosphatase, units/L; AST—aspartate aminotransferase, units/L; ALT—alanine aminotransferase, units/L; GGTP—gamma-glutamyl transpeptidase, units/L.
- Kidney function: CREAT—creatinine, mg/dL; BUN—blood urea nitrogen, mg/dL; SG—specific gravity; PH—urine pH; CCR—creatinine clearance rate = (140 − Age) × Weight/(Creatinine × 72) for Men OR ((140 − Age) × Weight/(Creatinine × 72)) × 0.85 for Women.
- Pancreas function: AMYLASE—amylase, units/L.
- Pulmonary function: FVC—forced lung capacity, measured in liters; FEV1—forced expiratory volume, measured in liters.

The study sample is representational regarding age. The number of men accounts for 60% of the total sample (Table 1).

Events of interest are ten cancer sites that are among the cancers with top incidence and top mortality rates: thyroid, gastric, breast, colorectal, lung, prostate, liver, kidney, uterine-cervical, and lymphoma cancers.

### 2.3. Project Pipeline

**Figure 1:**
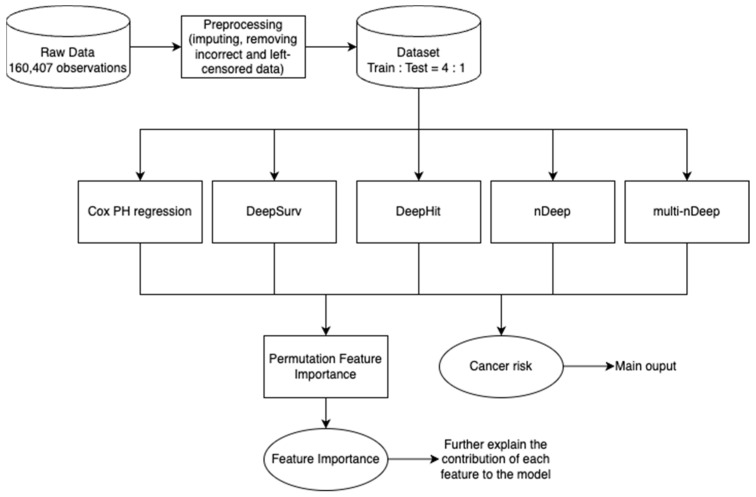
Project pipeline.

For the raw dataset, observations that are left-censored or contain values that are 6SD away from the mean were excluded (3.3% of the sample). Features with a missing rate of less than 25% were retained and imputed. The time variable was defined by subtracting the year of study enrolment from the year of getting diagnosed with any of the above cancers (event case) or finishing/quitting the study before any event (censored case). The processed data were then fed into each of the five models so that cancer risk and feature importance could be calculated (Figure 1). Our analysis process are released at https://github.com/ngocdung03/nDeep.

### 2.4. Model Description

Four neural networks and one Cox PH model were included for predictive performance comparison. 

#### 2.4.1. Cox PH Regression

Developed in 1972, Cox PH is a semi-parametric regression model for determining the association between predictors (covariates) and the rate of an event happening at a specific time point (hazard rate).

*h*(*t*) = *h*_0_(*t*)*exp*(*β* × *x_i_*)

where,

- *t* is the survival time
- *h*(*t*) is the hazard function
- *x_i_* is the vector of covariates
- *h*_0_(*t*) is the baseline hazard when all the xi are equal to zero.
- *β* is the vector of coefficients of *x_i_*

By maximizing this partial likelihood, the vector of *β* is estimated:

$$L_{c}(\;\beta )=\prod_{i:E_{i}=1}\frac{exp(\beta \times x_{i})}{\sum_{j\in R(T_{i})}exp(\beta \times x_{j})}$$

where,

- *E_i_*, *T_i_*, and *x_i_* are event indicator, survival time, and baseline covariates of the *i*th observation.
- The likelihood is defined in non-censored observations (*E_i_* = 1).
- *R*(*t*) = {*i*: *T_i_ >*= *t*} is the set of participants at risk of event at time *t*.

#### 2.4.2. DeepSurv

DeepSurv is a deep neural network (DNN) that outputs the estimate of hazard function $\hat{h_{\theta }}$(*x*) with *θ* as the weights of the network [9]. Unlike the model of [8], it applied modern techniques for training DNN, including weight decay regularization, batch normalization, Rectified Linear Units (ReLU), dropout, and gradient descent optimization. 

#### 2.4.3. DeepHit

DeepHit is a multitask neural network that is formed by shared layer(s) and K task-specific sub-networks (K is the number of tasks). There is a residual connection from input to each task-specific sub-network. Deep residual learning [14] is a technique for overcoming the accuracy degradation when increasing the depth of the network. In addition, with multitask learning, DeepHit can alleviate the competing risk issue, which is common in survival analysis and studies of cancer.

In this study, we performed two multitask learning DeepHit models on these groups of tasks: Group 1—thyroid, gastric, breast, colorectal, and lung cancers; and Group 2—prostate, liver, kidney, uterus-cervical, and lymphoma cancers. 

#### 2.4.4. nDeep

**Figure 2:**
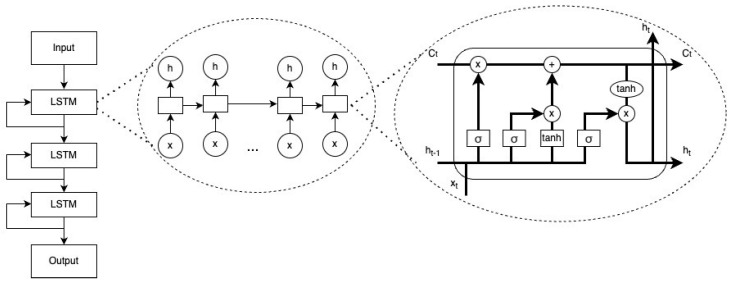
Architecture of nDeep.

This model contains three layers of long short-term memory (LSTM) and one fully connected layer that outputs predicted hazard. LSTM is a type of recurrent network (RNN) that has feedback connections that can address vanishing or exploding gradient issues of RNN [15]. Its architecture transforms survival data into sequences of n time steps, which can then be processed and analyzed. A basic LSTM unit includes a cell, an input gate, an output gate, and a forget gate (Figure 2). The equations for a forward pass of an LSTM cell are as follows:

$$f_{t}=\sigma_{g}\left(W_{f}x_{t}+U_{f}h_{t-1}+b_{f}\right)\;i_{t}=\sigma_{g}\left(W_{i}x_{t}+U_{i}h_{t-1}+b_{i}\right)\;o_{t}=\sigma_{g}\left(W_{o}x_{t}+U_{o}h_{t-1}+b_{o}\right)\;\tilde{c_{t}}=\sigma_{c}\left(W_{c}x_{t}+U_{c}h_{t-1}+b_{c}\right)\;c_{t}=f_{t}⊙c_{t-1}+i_{t}⊙\tilde{c_{t}}\;h_{t}=o_{t}⊙\sigma_{h}\left(c_{t}\right)$$

where,

- The initial values *c*_0_ = 0 and *h*_0_ = 0
- *x_t_* ∈ *R^d^*: vector of input
- *f_t_* ∈ (0, 1)*^h^*: vector of forget gate’s activation
- *i_t_* ∈ (0, 1)*^h^*: vector of input/update gate’s activation
- *o_t_* ∈ (0, 1)*^h^*: vector of output gate’s activation
- *h_t_* ∈ (−1, 1)*^h^*: vector of hidden state, also output
- $\tilde{c_{t}}$ ∈ (−1, 1)*^h^*: vector of cell input activation
- *c_t_* ∈ *R^h^*: vector of cell state
- *W* ∈ *R^hxd^*, *U* ∈ *R^hxd^* and *b* ∈ *R^h^*: vectors of weight matrices and bias
- ⊙: element-wise product (Hadamard product)

#### 2.4.5. Multi-nDeep

**Figure 3:**
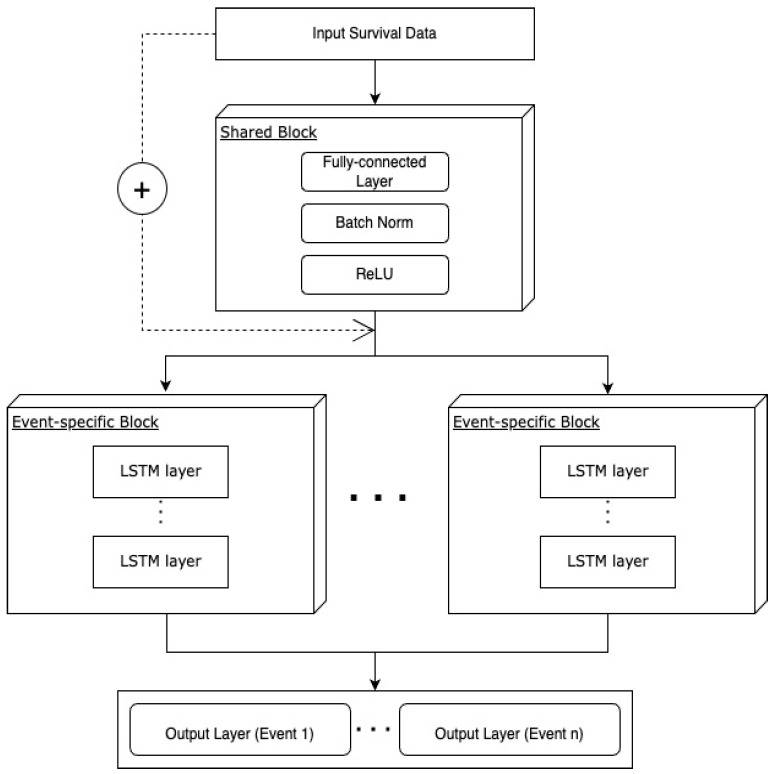
Architecture of mtl-nDeep.

Being inspired by the combination of multitask learning and residual connection, multi-nDeep has a shared block of fully connected layers and K task-specific blocks of nDeep network (Figure 3). The residual is connected from the input to each task-specific block. Models of multitask-nDeep were trained on these groups of tasks: Group 1—thyroid and gastric; Group 2—breast, colorectal, and lung; Group 3—prostate and liver; and Group 4—kidney, uterus-cervical, and lymphoma cancers.

#### 2.4.6. Hyperparameters of DNN Models

Hyperparameters of each DNN model are shown in Supplementary Table S1. Their optimization progress is depicted in Supplementary Figure S1.

### 2.5. Model Learning

**Figure 4:**
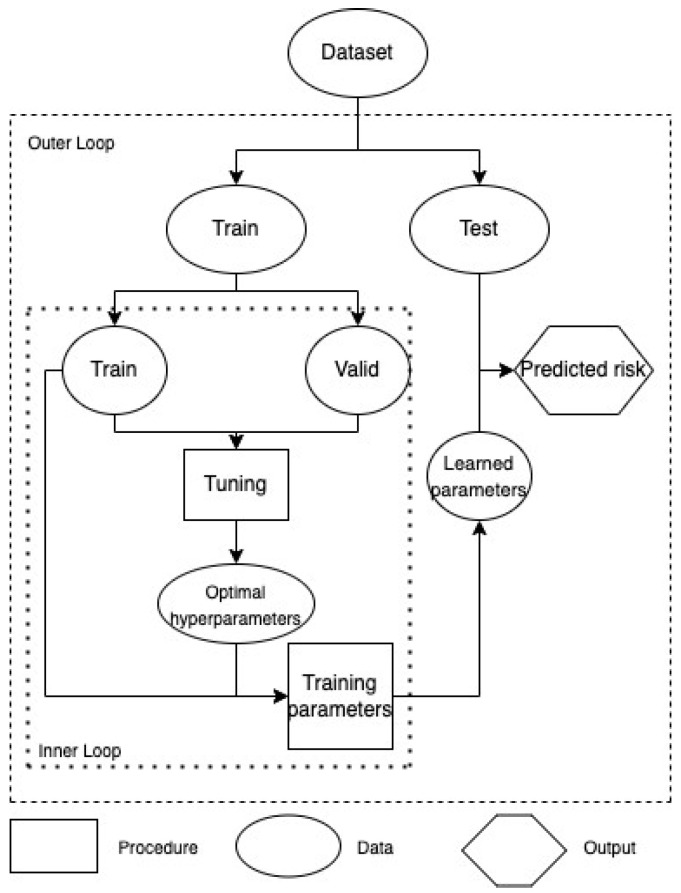
The workflow for optimizing hyperparameters of the models. The inner loop is a process to find the optimal hyperparameters during a training process. Furthermore, the outer loop is a process to evaluate the optimal hyperparameters that were obtained by training with test sets once again.

Four networks were involved in two training loops. The outer loop started with the dataset being randomly divided into a train set and a test set (ratio 4:1). In the inner loop, the valid set was further sampled on the train set with the probability of 0.2 so that hyperparameters were optimized by an automatic framework called Optuna with 60 iterations. This framework employs a Tree-structured Parzen Estimator (TPE), a state-of-the-art algorithm based on Bayesian optimization, which is faster and more efficient than grid search (Supplementary Figure S1). The tuned hyperparameters were used for training parameters of each neural network. The inner loop returned a learned model, which was run on the test set 30 times to output predicted cancer hazard risks and averaged c-index (Figure 4).

The loss function used for each DNN is calculated by the sum of L1 + L2 where: L1 is the negative log partial likelihood:

$$L1=-\sum_{i=1}^{N}I_{i}\left[\hat{h_{\theta }}\left(x_{i}\right)-log\sum_{j\in R\left(t_{i}\right)}e^{\hat{h_{\theta }}\left(x_{j}\right)}\right]$$

where,

- *N*: number of subjects
- *I*: indicator function (1: death, 0: otherwise)
- *θ*: weights of the network
- $\hat{h_{\theta }}$: estimated hazard function
- *x_i_, x_j_*: vectors of covariates
- *R*(*t_i_*): set of subjects at risk at time *t_i_*

L2 is the ranking loss function:

$$L2=\sum_{i\neq j}I_{i,j}.\eta (\hat{F}(s^{\left(i\right)}|x^{\left(i\right)}),\;\hat{F}(s^{\left(j\right)}|x^{\left(j\right)})$$

where,

- *I_i,j_*: indicator function of acceptable pairs (*i*,*j*) [16]
- $\eta \left(x,y\right)=exp\left(\frac{-\left(x-y\right)}{\sigma }\right)$: convex loss function
- $\hat{F}$: estimated cumulative incidence function
- *s*: time
- *x*: covariate

### 2.6. Performance Metric

Model accuracy was evaluated by Harrell’s concordance index (c-index) [16]. A c-index of 1 represents perfect accuracy. Contrary, a c-index which is 0.5 or less suggests the model performs no better than a naïve model. Five-fold cross-validation was performed 30 times for each task, with the test set being sampled with replacement. The final c-index was achieved by averaging the c-indices of those 30 trials.

### 2.7. Feature Importance

For each model, permutation feature importance (PFI) was calculated by the module permutation importance of *sklearn* version 1.0.2—a Python library. PFI scores were evaluated based on the c-index. The values of PFI are presented in figures and grouped by a separation line for interpretation. The groups of features represent demographic, metabolic, liver function, pulmonary function, kidney function, and pancreas function.

### 2.8. Ethical Considerations

Informed consent of participants was received under the support of the Metabolic Syndrome Research Program of the Seoul Metropolitan Government in December 2005. This study was approved by the Yonsei University Institutional Review Board (IRB Approval No. 4-2011-0277).

The current study is approved by the Korea Institute of Bioethics Policy Electromagnetic Concern Committee to be exempt from examination. All methods were used based on ethical regulations and guidelines.

## 3. Results

Table 2 indicates the number of cases on each cancer site and the corresponding rate over the total number of samples. Cases/total sample rates were small, suggesting an imbalance in data.

### 3.1. Comparing c-Index of Each Model

**Figure 5:**
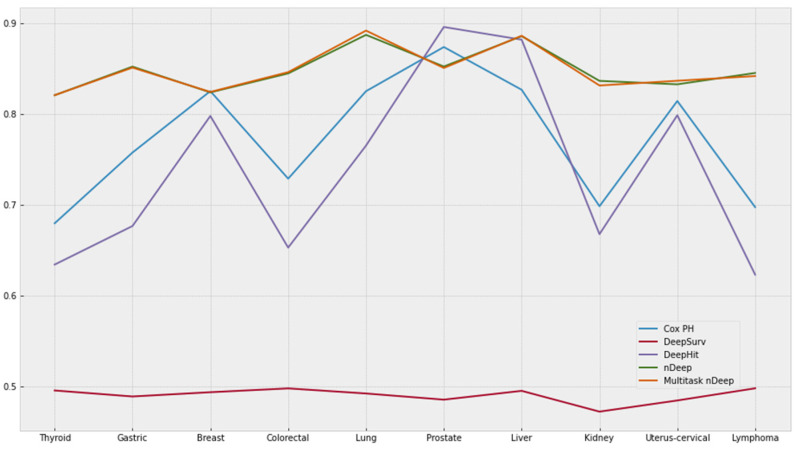
Best c-index of each model. The y-axis represents c-index values.

Figure 5 shows the c-index of five models on different tasks of predicting cancer. DeepHit and Cox PH regression models attained the equivalent pattern of c-index. Although c-indices of DeepHit on many cancers are slightly lower as compared to Cox PH, DeepHit, a multitasking model, reached c-index as high as 0.8962 and 0.8822 for prostate and liver cancers. DeepSurv did not indicate any predictive power with the c-index of about 0.5 for every model. On the other hand, nDeep and multi-nDeep achieved the c-indices, which are consistently more than 0.8. Although there was a slight improvement in multi-nDeep as compared to nDeep, the effects were not remarkable. In Supplementary Table S2, the best c-index of each cancer is presented in bold. For reference purpose, c-indices of variants of Cox PH were included in Table S3.

### 3.2. Feature Importance

**Figure 6:**
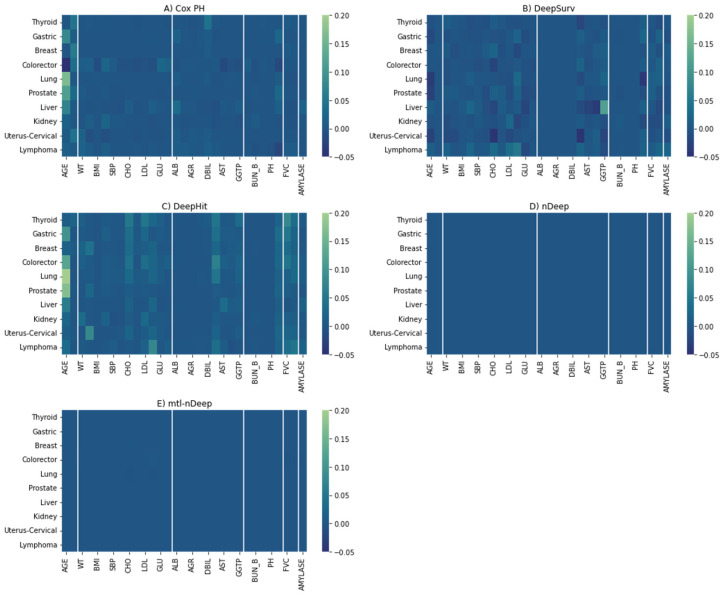
The heatmaps of PFI for five models. Differences in the c-index after removing a feature were represented in a manner where the brighter the color, the more important the feature is. (**A**) Cox PH. (**B**) DeepSurv. (**C**) DeepHit. (**D**) nDeep. (**E**) mtl-nDeep.

For Cox PH, the most important features are demographic ones (Age and Sex), followed by liver function (Figure 6A). Age is the most decisive factor in the majority of Cox PH models, while sex applies to thyroid cancer, breast cancer, colorectal, and uterus-cervical cancers (particularly, women have a higher risk of these cancers as compared with men in this study). In addition, direct bilirubin, a liver function metric, was also a crucial determinant of thyroid cancer. Metabolic characteristics even showed higher importance than demographic factors for kidney and lymphoma cancers, where waist circumference and weight were the main predictors. Kidney function characteristics showed moderate importance for some cancers (creatinine clearance rate for gastric, prostate, and liver cancers, and blood urea nitrogen for kidney cancer). Amylase, a pancreas function index, and creatinine clearance rate were moderately important for liver cancer.

Similar patterns were observed for DeepHit on gastric, lung, prostate, liver, kidney, and lymphoma cancers, although there were more contributions from factors across categories other than the dominant factor (Figure 6C). For kidney and lymphoma cancer, metabolic characteristics were still the most important but attributed to other metrics (weight and low-density lipoprotein, and triglyceride, respectively). Regarding colorectal cancer, age is the main determinant rather than sex. PFIs, according to thyroid and breast cancers, are relatively equitably distributed across categories. Uterus-cervical cancer is mostly attributed to height.

The figure of feature importance for DeepSurv (Figure 6B) was almost uninformative, with rather random distributions around zero. In Figure 6D,E, with PFI score <0.001, the effect of each feature on nDeep models was minor. Generally, the patterns of multitask-nDeep were similar to nDeep.

## 4. Discussion

Only two models, nDeep and multitask nDeep, outperformed the traditional Cox PH regression model. On the other hand, DeepSurv exhibited no improvement over a naïve model. This is inconsistent with the results presented in reference [9], where the c-index of DeepSurv can be more than 0.7. Similarly, DeepHit showed comparable c-indices with Cox PH in our study, while its performance was significantly better in generated data [10]. That signifies the disparities in nature between the simulated and real censored data. One of the persistent challenges in follow-up health data is the low rate of uncensored cases, especially when the event is a rare disease such as cancer, which poses significant hurdles for the accuracy of survival analysis [17]. Furthermore, in our study, DeepSurv is the only neural network without residual connections, which could explain its underperformance. In contrast, other than the well-known Cox PH, nDeep emerges as the best model in terms of both accuracy and cost-effectiveness in the current study. It seems that the absence of a particular feature did not hinder the performance of nDeep and its variants.

There are two main factors that affect nDeep performance. Firstly, drawing inspiration from natural language processing, we treated the observed data for a participant and a visit as a single sequence with n time steps and one subsequence with m time steps (where m < n), respectively. Secondly, our model architectures incorporated the benefits of residual connections and multiple gates in an LSTM memory cell to effectively capture the dynamic nature of covariates. Moreover, finding the optimal models was eased by applying techniques including TPE algorithm.

The connection between age and cancer is well-established [18]. In fact, our analysis based on Cox PH regression models indicates that age has an impact on nearly all types of cancers. Aging decreases the effectiveness of autophagy, a process responsible for damaged organelles removal, leading to uncontrollable cellular homeostasis and cancer genesis [19]. Some cancers, such as breast and uterus-cervical cancers, are strongly affected by sex, which is expected as these cancers predominantly or exclusively affect females [2,12]. Additionally, our study reveals a higher prevalence of colorectal cancer in women compared to men. This is possibly due to both genetic and environmental factors, including dietary habits [20]. Similarly, the female predominance in thyroid cancer cases can be attributed to factors such as sex hormones, genetics, and the immune system [21,22,23,24].

Neural network like DeepHit helps learn the non-linear effects of factors beyond age and sex in cancer risk assessment. It was observed that metabolic metrics, liver function, pulmonary function, and kidney function indices serve as predictors for certain cancer types, including thyroid, gastric, breast, colorectal, kidney, and lymphoma cancers.

Obesity-related metabolic disorders have long been recognized as risk factors for thyroid cancer [25]. Specifically, WC is a strong indicator of the association between overweight/obesity and thyroid cancer [26]. Obesity also highly promotes the progression of various other cancers through mechanisms involving hormones, insulin and insulin-like growth factors, sex steroids, and obese inflammation [27,28]. Increased inflammation in adipose tissue, a consequence of obesity, results in changes in adipokine secretion patterns and the release of pro-inflammatory cytokines, leading to insulin resistance rises in tissues with active metabolism [29]. Overexpression of insulin-like growth factors may have neoplastic effects by promoting cell cycle progression and inhibiting apoptosis, either directly or indirectly, through interaction with established oncogenic systems such as steroid hormones and integrins [30]. Moreover, excess peripheral adipose tissue contributes to steroid aromatization, leading to elevated estrogen levels. Androgens and androgenic antecedents are changed over to estradiol by the chemical aromatase. Within the setting of weight and abundance of fat tissue, aromatase expanded action leads to higher transformation rates coming about in higher levels of estrogens [31]. Estrogen advances tumorigenesis in endometrial tissue by incitement of cell multiplication and hindrance of apoptosis [32].

A recent study demonstrated a link between metabolic dysfunction-associated fatty liver and thyroid cancer, even after adjusting for WC [33]. The relationship between thyroid cancer, a hormone-sensitive cancer, and metabolic dysfunction was found to be modified by menopausal status in females. Higher levels of liver function markers were pointed out to be associated with a reduced risk of colorectal cancer [34]. In the ileum, bilirubin undergoes deconjugation through bacterial and mucosal processes, potentially possessing antioxidant and anti-inflammatory properties [35]. Considering that inflammation and oxidative stress are pivotal contributors to cancer development, this association is noteworthy [36]. The link between pulmonary function and cancer is primarily limited to lung cancer [37]. Likewise, kidney function has been found to be associated with kidney cancer mainly.

This study represents the first attempt to compare different neural network models and traditional models in survival analysis using clinical biomarkers on a large-scale cohort. Our findings align with prior evidence indicating that deep neural networks outperform other methods in healthcare survival analysis. Furthermore, we have clarified the role of different factors in predicting cancer incidence. However, according to the PFI heatmaps, while Cox PH regression heavily relies on information regarding sex and age, cancers appear to be more evenly affected by all the features in our models. Therefore, in the future, we should investigate advanced models capable of distinguishing key factors clearly and incorporate additional biomarkers for more accurate cancer prediction. Additionally, exploring the causal relationship between key factors and specific cancers is crucial, as it can help identify causal factors and facilitate cancer prevention efforts. 

## 5. Conclusions

We have introduced nDeep and its multitasking version for predicting cancer risks based on clinical biomarkers. These algorithms transform health follow-up data into sequences that can be fed into recurrent neural networks, enabling high predictive performance. Our model can assist health care providers in determining people at risk of ten different cancers. 

## Figures and Tables

**Table 1:** Basic information on demography, anthropometry, and clinical characteristics of study population.

	Mean ± SD	Min	Max	Male Mean ± SD	Female Mean ± SD
AGE	41.62 ± 10.53	9.00	88.00	42.04 ± 9.95	40.98 ± 11.31
HT	166.80 ± 8.40	130.00	198.00	171.75 ± 5.95	159.31 ± 5.52
WT	65.79 ± 12.00	32.00	137.00	72.12 ± 9.93	56.23 ± 7.78
WC	80.58 ± 9.45	41.00	127.00	84.80 ± 7.65	74.21 ± 8.26
BMI	23.53 ± 3.17	12.91	42.36	24.42 ± 2.88	22.18 ± 3.10
SBP	117.65 ± 14.11	61.00	19 9.00	121.23 ± 12.87	112.25 ± 14.19
DBP	74.12 ± 10.04	30.00	134.00	76.55 ± 9.56	70.45 ± 9.63
CHO	188.83 ± 33.02	3.68	382.00	192.04 ± 32.86	183.99 ± 32.68
HDL	52.27 ± 10.70	2.00	118.00	48.80 ± 9.01	57.50 ± 10.92
TG	132.82 ± 83.35	30.00	690.00	155.29 ± 90.44	98.96 ± 56.51
LDL	112.13 ± 31.03	1.00	299.60	115.23 ± 31.25	107.47 ± 30.11
FVC	26.93 ± 30.82	0.04	158.00	25.33 ± 30.39	29.16 ± 31.33
FEV1	28.64 ± 33.96	0.42	178.00	26.58 ± 33.25	31.56 ± 34.81
ALB	4.53 ± 0.25	2.20	5.90	4.58 ± 0.24	4.45 ± 0.24
GLOBULIN	2.76 ± 0.17	1.80	3.80	2.74 ± 0.17	2.78 ± 0.16
AGR	1.64 ± 0.13	0.85	2.39	1.67 ± 0.13	1.59 ± 0.11
BIL	0.87 ± 0.33	0.10	2.93	0.94 ± 0.34	0.75 ± 0.28
DBIL	0.33 ± 0.12	0.04	1.07	0.36 ± 0.12	0.28 ± 0.10
ALP	133.52 ± 50.56	10.00	445.00	142.70 ± 50.77	119.69 ± 46.93
AST	22.69 ± 8.90	1.00	135.00	24.65 ± 9.55	19.75 ± 6.85
ALT	24.43 ± 17.23	1.00	180.00	29.46 ± 18.95	16.87 ± 10.68
GGTP	34.23 ± 32.45	1.90	390.00	44.58 ± 36.75	18.67 ± 14.83
GLU	90.56 ± 15.19	10.00	208.00	92.37 ± 16.18	87.83 ± 13.07
AMYLASE	70.98 ± 19.00	3.00	195.00	69.63 ± 18.65	73.04 ± 19.35
CREAT	0.98 ± 0.18	0.38	2.40	1.09 ± 0.14	0.83 ± 0.12
BUN	13.74 ± 3.25	0.45	59.00	14.50 ± 3.13	12.57 ± 3.07
SG	1.02 ± 0.01	1.00	1.05	1.02 ± 0.01	1.02 ± 0.01
PH	5.68 ± 0.76	0.50	9.00	5.66 ± 0.75	5.72 ± 0.77
PP	43.54 ± 9.64	0.00	101.00	44.69 ± 9.50	41.80 ± 9.59
CCR	92.92 ± 20.02	20.14	213.37	91.66 ± 19.86	94.85 ± 20.12

**Table 2:** Number and rate of cancer cases on each site.

Cancer Site	Number of Cases	Cases/Total Cancer Cases	Cases/Total Sample
Thyroid	3385	0.33120	0.02189
Gastric	1499	0.15109	0.00969
Breast	1169	0.11783	0.00756
Colorectum	1031	0.10392	0.00667
Lung	757	0.07630	0.00489
Prostate	697	0.07026	0.00451
Liver	439	0.04425	0.00284
Kidney	352	0.03548	0.00228
Uterus-cervical	306	0.03084	0.00198
Lymphoma	286	0.02883	0.00185

## Data Availability

The raw data supporting the conclusions of this article will be made available by the authors, without undue reservation.

## Supplementary Materials

The following supporting information can be downloaded at: https://www.mdpi.com/article/10.3390/cancers15194757/s1, Figure S1: Hyperparameters optimization of 4 models bases on TPE algorithms; Table S1: Hyperparameters of DNN models; Table S2: C-index of each model and imputation method; Table S3: C-index of variants of Cox PH models.
