# Supplementary material for: A Study on Survival Analysis Methods Using Neural Network to Prevent Cancers
Source: Cancers (Basel). 2023 Sep 27;15(19):4757. doi: 10.3390/cancers15194757 (PMC10571885; doi:10.3390/cancers15194757)
Supplement: Supplementary file 1 [file cancers-15-04757-s001.zip › cancers-2596833-supplementary.pdf]

Table S1. Hyperparameters of DNN models. Description and range of each hyperparameter: learning rate - Beginning learning rate (1e-5 - 1e-1) ; optimizer - Optimization method (Adam, RMSprop); epoch - Number of trials of inner loop/epoch (1-10 for DeepSurv and DeepHit, 1-4 for nDeep and Multi-nDeep) ; p dropout - Rate of dropping out the nodes (0.0 - 0.8).

<table>
<tr><th>Model</th><th>Hypeparameters</th><th>Thyroid</th><th>Gastric</th><th>Breast</th><th>Colorectal</th><th>Lung</th><th>Prostate</th><th>Liver</th><th>Kidney</th><th>Uterus-Cervical</th><th>Lymphoma</th></tr>
<tr><td rowspan="4">DeepSurv</td><td>learning_rate</td><td>0.041920202</td><td>0.000118351</td><td>0.004134312</td><td>0.000151401</td><td>0.003950026</td><td>0.000836143</td><td>5.32E-05</td><td>0.099151413</td><td>7.15E-05</td><td>0.08306069</td></tr>
<tr><td>optimizer</td><td>RMSprop</td><td>Adam</td><td>Adam</td><td>Adam</td><td>Adam</td><td>Adam</td><td>Adam</td><td>Adam</td><td>Adam</td><td>Adam</td></tr>
<tr><td>n_epochs</td><td>10</td><td>9</td><td>10</td><td>10</td><td>4</td><td>5</td><td>6</td><td>4</td><td>3</td><td>6</td></tr>
<tr><td>p_dropout</td><td>0.183634881</td><td>0.31973134</td><td>0.094106354</td><td>0.206362516</td><td>0.539981643</td><td>0.253549335</td><td>0.043863264</td><td>0.788817713</td><td>0.775532345</td><td>0.707465165</td></tr>
<tr><td rowspan="4">DeepHit</td><td>learning_rate</td><td colspan="5">0.001096384</td><td colspan="5">7.55E-05</td></tr>
<tr><td>optimizer</td><td colspan="5">Adam</td><td colspan="5">Adam</td></tr>
<tr><td>n_epochs</td><td colspan="5">8</td><td colspan="5">7</td></tr>
<tr><td>p_dropout</td><td colspan="5">0.601865894</td><td colspan="5">0.053397595</td></tr>
<tr><td rowspan="4">nDeep</td><td>learning_rate</td><td>0.001887609</td><td>0.001385036</td><td>0.038016289</td><td>0.001925324</td><td>0.000811696</td><td>0.008291019</td><td>0.000639385</td><td>0.004502712</td><td>0.003476174</td><td>0.00099045</td></tr>
<tr><td>optimizer</td><td>RMSprop</td><td>RMSprop</td><td>RMSprop</td><td>RMSprop</td><td>RMSprop</td><td>Adam</td><td>RMSprop</td><td>Adam</td><td>RMSprop</td><td>RMSprop</td></tr>
<tr><td>n_epochs</td><td>4</td><td>3</td><td>4</td><td>3</td><td>3</td><td>4</td><td>3</td><td>3</td><td>3</td><td>3</td></tr>
<tr><td>p_dropout</td><td>0.007023522</td><td>0.008543556</td><td>0.055343576</td><td>0.167942658</td><td>0.002501462</td><td>0.000163165</td><td>0.002007299</td><td>0.000334646</td><td>0.003730368</td><td>0.005387184</td></tr>
<tr><td rowspan="4">Multi-nDeep</td><td>learning_rate</td><td colspan="2">0.004059415</td><td colspan="3">0.015921436</td><td colspan="2">0.090245464</td><td colspan="3">0.000485033</td></tr>
<tr><td>optimizer</td><td colspan="2">Adam</td><td colspan="3">RMSprop</td><td colspan="2">Adam</td><td colspan="3">RMSprop</td></tr>
<tr><td>n_epochs</td><td colspan="2">4</td><td colspan="3">2</td><td colspan="2">4</td><td colspan="3">3</td></tr>
<tr><td>p_dropout</td><td colspan="2">0.039516671</td><td colspan="3">0.003251124</td><td colspan="2">0.240899609</td><td colspan="3">0.208187035</td></tr>
</table>

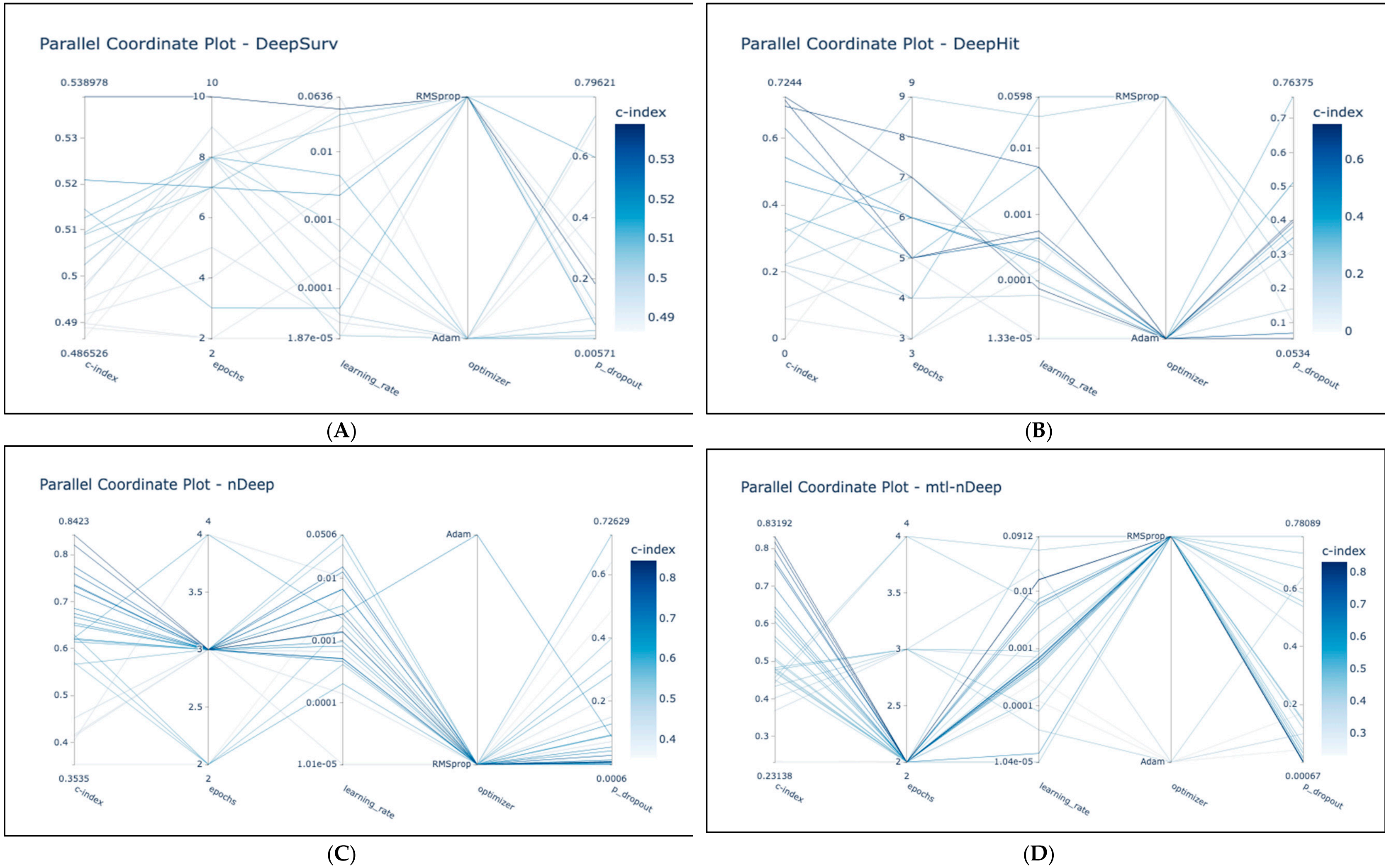

Figure S1. Hyperparameters optimization of 4 models bases on TPE algorithms. (**A**) DeepSurv on thyroid cancer; (**B**) DeepHit on prostate, liver, kidney, uterus-cervical, and lymphoma cancer; (**C**) nDeep on gastric cancer; (**D**) Multi-nDeep on breast, colorectal, and lung cancer. For multitask-learning models (Multi-nDeep and DeepHit), the mean c-index among tasks was used as the criteria for hyperparameter tuning. As can be shown, the parallel coordinate plots of nDeep and Multi-nDeep are more convergent than those of DeepSurv and DeepHit.

Table S2. C-index of each model and imputation method.

Model	Target	Imputation by mean		Imputation by median		Imputation by regression	
		Test	Test-cases only	Test	Test-cases only	Test	Test-cases only
Cox PH	Thyroid	0.6752	0.6171	0.6667	0.6197	0.6798	0.6656
	Gastric	0.7560	0.6889	0.7578	0.6797	0.7574	0.5739
	Breast	0.8250	0.7941	0.8177	0.8020	0.8255	0.7008
	Colorectal	0.7290	0.5987	0.7152	0.5581	0.7267	0.5563
	Lung	0.8247	0.6629	0.8254	0.6215	0.8248	0.5669
	Prostate	0.8741	0.8066	0.8632	0.7862	0.7197	0.6107
	Liver	0.8270	0.5189	0.8268	0.5020	0.6896	0.5003
	Kidney	0.6986	0.6931	0.6941	0.5991	0.5724	0.5583
	Uterine-cervical	0.8146	0.8167	0.8105	0.7897	0.6694	0.7497
	Lymphoma	0.6976	0.5957	0.6887	0.5523	0.6268	0.5119
DeepSurv	Thyroid	0.4955	0.4996	0.4518	0.4731	0.4713	0.4966
	Gastric	0.4614	0.4754	0.4889	0.4794	0.4492	0.4888
	Breast	0.4816	0.4788	0.4576	0.4737	0.4936	0.4844
	Colorectal	0.4813	0.4863	0.4978	0.4897	0.4756	0.4983
	Lung	0.4739	0.4903	0.4922	0.4853	0.4441	0.4971
	Prostate	0.4739	0.4966	0.4854	0.4793	0.4390	0.4883
	Liver	0.4951	0.4907	0.4590	0.4758	0.4500	0.4747
	Kidney	0.4687	0.4880	0.4722	0.4794	0.4689	0.4807
	Uterine-cervical	0.4810	0.4767	0.4471	0.4836	0.4845	0.4826
	Lymphoma	0.4741	0.4900	0.4514	0.4908	0.4979	0.4739
DeepHit	Thyroid	0.6304	0.6966	0.6343	0.6430	0.6115	0.6721
	Gastric	0.6682	0.6583	0.6768	0.6321	0.6187	0.6006
	Breast	0.7949	0.6140	0.7981	0.5705	0.7303	0.5752
	Colorectal	0.6530	0.5569	0.6511	0.5884	0.5967	0.5262
	Lung	0.7485	0.6574	0.7654	0.5765	0.6845	0.5724
	Prostate	**0.8962**	0.7300	0.8957	0.7502	0.8653	0.7109
	Liver	0.8822	0.7418	0.8767	0.7203	0.8196	0.7366
	Kidney	0.6619	0.6271	0.6678	0.7000	0.6456	0.6659

Model	Target	Imputation by mean		Imputation by median		Imputation by regression	
		Test	Test-cases only	Test	Test-cases only	Test	Test-cases only
	Uterine-cervical	0.7989	0.5958	0.7959	0.6073	0.7627	0.5828
	Lymphoma	0.6232	0.7254	0.6197	0.6468	0.5922	0.6678
nDeep	Thyroid	0.8201	0.8459	0.8210	0.8468	0.8185	0.8462
	Gastric	0.8506	0.8609	**0.8525**	0.8708	0.8517	0.8604
	Breast	0.8240	0.8436	0.8241	0.8332	0.8226	0.8439
	Colorectal	0.8418	0.8649	0.8450	0.8513	0.8427	0.8484
	Lung	0.8875	0.8893	0.8869	0.8870	0.8862	0.8901
	Prostate	0.8509	0.8694	0.8506	0.8436	0.8526	0.8626
	Liver	0.8838	0.8879	0.8861	0.8934	0.8844	0.8869
	Kidney	**0.**8368	0.8334	0.8327	0.8552	0.8264	0.8330
	Uterine-cervical	0.8330	0.8494	0.8259	0.8476	0.8306	0.8456
	Lymphoma	0.8433	0.8487	0.8415	0.8531	**0.8455**	0.8591
Multitask nDeep	Thyroid	0.8201	0.8459	0.8208	0.8468	**0.8210**	0.8458
	Gastric	0.8511	0.8609	0.8474	0.8708	0.8514	0.8605
	Breast	**0.8246**	0.8430	0.8229	0.8325	0.8197	0.8422
	Colorectal	0.8411	0.8643	0.8449	0.8524	**0.8464**	0.8493
	Lung	**0.**8922	0.8978	0.8858	0.8868	0.8867	0.8913
	Prostate	0.8510	0.8696	0.8498	0.8436	0.8478	0.8626
	Liver	0.8856	0.9086	**0.8865**	0.9088	0.8848	0.8797
	Kidney	0.8279	0.8334	0.8302	0.8552	0.8317	0.8284
	Uterine-cervical	0.8327	0.8494	**0.8369**	0.8476	0.8328	0.8444
	Lymphoma	0.8421	0.8487	0.8352	0.8531	0.8404	0.8565

Table S3. C-index of variants of Cox PH models.

Model	Target	Imputation by mean	Imputation by median	Imputation by regression
L1 regularized-Cox PH	Thyroid	0.6009	0.6045	0.6045
	Gastric	0.6523	0.6607	0.6624
	Breast	0.7002	0.6977	0.7236
	Colorectal	0.6705	0.6226	0.6558
	Lung	0.7139	0.7473	0.7566
	Prostate	0.8518	0.8248	0.8161
	Liver	0.8297	0.8286	0.8279
	Kidney	0.5801	0.5861	0.6514
	Uterine-cervical	0.7300	0.7061	0.6900
	Lymphoma	0.6496	0.6791	0.5500
L2 regularized-Cox PH	Thyroid	0.6179	0.6535	0.6613
	Gastric	0.7325	0.6714	0.5929
	Breast	0.7709	0.8147	0.7715
	Colorectal	0.7315	0.5773	0.5949
	Lung	0.7826	0.7272	0.4760
	Prostate	0.8847	0.8614	0.6117
	Liver	0.8264	0.5624	0.4889
	Kidney	0.6689	0.6760	0.5498
	Uterine-cervical	0.8285	0.8360	0.6483
	Lymphoma	0.6832	0.6169	0.6293
